# Assessment of All-Cause Cancer Incidence Among Individuals With Preeclampsia or Eclampsia During First Pregnancy

**DOI:** 10.1001/jamanetworkopen.2021.14486

**Published:** 2021-06-23

**Authors:** Chris Serrand, Thibault Mura, Pascale Fabbro-Peray, Gilles Seni, Ève Mousty, Thierry Boudemaghe, Jean-Christophe Gris

**Affiliations:** 1 Department of Biostatistics, Clinical Epidemiology, Public Health, and Innovation in Methodology, Centre Hospitalier Universitaire de Nîmes, Groupe Hospitalo–Universitaire Caremeau, Nîmes, France; 2 Faculty of Medicine, University of Montpellier, Montpellier, France; 3 Institut National de la Santé et de la Recherche Médicale UA11, Institut Desbrest d’Épidémiologie et de Santé Publique, University of Montpellier, Montpellier, France; 4 Department of Medical Information, Methods and Research, Centre Hospitalier Universitaire de Nîmes, University of Montpellier, Nîmes, France; 5 Department of Gynecology and Obstetrics, Centre Hospitalier Universitaire de Nîmes, University of Montpellier, Nîmes, France; 6 Department of Hematology, Centre Hospitalier Universitaire de Nîmes, University of Montpellier, Nîmes, France; 7 Faculty of Pharmaceutical and Biological Sciences, University of Montpellier, Montpellier, France; 8 I.M. Sechenov First Moscow State Medical University, Moscow, Russian Federation

## Abstract

**Importance:**

Preeclampsia or eclampsia (preeclampsia/eclampsia) during pregnancy induces major physiological changes and may be associated with specific cancer occurrences in later life. The current data regarding the association between preeclampsia/eclampsia and cancer are heterogeneous, and cancer risk after preeclampsia/eclampsia could be different depending on the organ. These uncertainties warrant reexamination of the association between preeclampsia/eclampsia and the risk of cancer overall and by specific cancer type.

**Objective:**

To evaluate the risk of cancer, overall and by type, after preeclampsia/eclampsia during a first pregnancy.

**Design, Setting, and Participants:**

This retrospective cohort study used data from the French hospital discharge database to identify all female individuals who had a pregnancy-associated hospitalization between January 1, 2010, and December 31, 2019. To allow a minimum of 2 years for the detection of medical history, individuals with a first detected pregnancy before January 1, 2012, were excluded, as were those with a cancer-associated hospitalization before or during their first detected pregnancy. Exposures, comorbidities, and occurrences of cancer were evaluated using data from the medico-administrative registers of hospitalizations in private and public French hospitals. Cox proportional hazards models were used to analyze cancer risk according to the occurrence of preeclampsia/eclampsia during first pregnancy.

**Exposures:**

Preeclampsia/eclampsia-associated hospitalization during the first detected pregnancy.

**Main Outcomes and Measures:**

The primary outcome was the incidence of cancer, including myelodysplastic or myeloproliferative diseases, after a first pregnancy with and without preeclampsia/eclampsia.

**Results:**

After exclusions, a total of 4 322 970 female individuals (mean [SD] age at first detected pregnancy, 29.6 [6.2] years) with and without preeclampsia/eclampsia during their first pregnancy were included. Of those, 45 523 individuals (1.1%) were diagnosed with preeclampsia/eclampsia during their first detected pregnancy. The maximum follow-up was 8 years, during which 29 173 individuals (0.7%) were diagnosed with cancer. No significant difference in overall cancer incidence was found between those with and without preeclampsia/eclampsia during their first pregnancy (adjusted hazard ratio [AHR], 0.94; 95% CI, 0.84-1.05). Preeclampsia/eclampsia was associated with an increase in the risk of myelodysplastic syndromes or myeloproliferative diseases (AHR, 2.43; 95% CI, 1.46-4.06) and kidney cancer (AHR, 2.19; 95% CI, 1.09-4.42) and a decrease in the risk of breast cancer (AHR, 0.79; 95% CI, 0.62-0.99) and cervical cancer (AHR, 0.75; 95% CI, 0.58-0.96).

**Conclusions and Relevance:**

In this study, a history of preeclampsia/eclampsia during first pregnancy was associated with an increase in the incidence of myelodysplastic or myeloproliferative diseases and kidney cancer and a decrease in the incidence of cervical and breast cancers. These associations might reflect an underlying common factor among preeclampsia/eclampsia and these pathologies and/or an association between preeclampsia/eclampsia and the development of these cancers.

## Key Points

**Question:**

Is the occurrence of preeclampsia or eclampsia during a first pregnancy associated with a future risk of cancer?

**Findings:**

In this cohort study of 4 322 970 female individuals with and without preeclampsia or eclampsia during their first pregnancy, myelodysplastic syndromes or myeloproliferative diseases and kidney cancer were more common among those who experienced preeclampsia or eclampsia, whereas breast cancer was less common.

**Meaning:**

The study’s findings suggest that there may be a pathophysiological association between preeclampsia or eclampsia during a first pregnancy and the incidence of some myelodysplastic syndromes or myeloproliferative diseases, kidney cancer, and breast cancer.

## Teaser

This cohort study uses data from the French hospital discharge database to assess the association between preeclampsia or eclampsia during first pregnancy and the risk of future cancer and some cancer-associated blood diseases among female participants with a first pregnancy between 2012 and 2019.

## Introduction

Preeclampsia, the prodromal syndrome of eclampsia, is a multisystem syndrome that is diagnosed in 2% to 5% of pregnancies.^1^ It is defined by the presence of pregnancy-induced hypertension and proteinuria that appear after 20 weeks of amenorrhea. Preeclampsia or eclampsia (preeclampsia/eclampsia) is the most common syndrome within a spectrum of late pregnancy complications (referred to as placenta-mediated complications or placental syndromes) that includes fetal growth restriction, abruptio placenta, late pregnancy loss or intrauterine fetal death, and premature delivery associated with disordered placentation.^2,3,4,5^

The physiological demands of pregnancy have been viewed as a maternal stress test that can estimate health in later life.^6^ An increased death rate among those with a history of hypertension during pregnancy has been reported.^7^ A systematic review and meta-analysis reported an increased risk of vascular disease among those with preeclampsia/eclampsia but no increased risk of cancer in later life.^8^ One possible explanation for this finding is that the risk of each type of cancer was not analyzed.

Several factors suggest that there may be a change in the risk of some types of cancer after preeclampsia/eclampsia. First, preeclampsia/eclampsia induces hormonal surges and disorders that alter hormonal levels during pregnancy, which might be associated with the subsequent risk of hormone-dependent cancers.^9^ Second, a prevailing feature of preeclampsia/eclampsia is antiangiogenesis,^3^ which is essential to restricting tumor growth^10^; thus, preeclampsia/eclampsia may be associated with a reduction in the risk of solid tumors in later life. Consistent with this hypothesis, a reduced risk of breast cancer among female individuals with a history of preeclampsia/eclampsia has been described,^9^ particularly among those who are premenopausal and those who have breast cancer associated with the ERB-B2 receptor tyrosine kinase 2 (*ERBB2*; formerly human epidermal growth factor receptor 2 [*HER2*]) gene.^11,12,13^ Another study reported an increased risk of endometrial cancer.^14^ The conclusions concerning the existence and direction of a possible association between preeclampsia/eclampsia and cancer are therefore heterogeneous, and cancer risk after preeclampsia/eclampsia could be different depending on the organ.^15,16,17,18^ These uncertainties warrant reexamination of the association between preeclampsia/eclampsia and the short- to medium-term risk of a cancer diagnosis overall and by specific cancer type.

## Methods

### Study Design and Data Source

This retrospective cohort study used data from the French hospital discharge database to identify all female individuals with a recorded pregnancy between January 1, 2010, and December 31, 2019. This database records all inpatient hospital stays in private and public hospitals in a standardized data set.^19^ In France, more than 99% of deliveries occur in maternity hospitals and are therefore recorded.^20^ This study used hospitalization data that are available to all teaching hospitals for research purposes. The French hospital discharge database is limited to medico-administrative information and can be used to assess the health status of populations without the need for institutional review board approval in France with the condition that researchers respect a set of ethical rules regarding data handling. This study followed the Strengthening the Reporting of Observational Studies in Epidemiology (STROBE) reporting guideline for cohort studies.

The available data included age, sex, hospital admission and discharge dates, diagnostic codes (according to the *International Classification of Diseases, Tenth Revision* [*ICD-10*]) associated with hospitalizations, insurance coverage (state-issued or private), and deaths that occurred during hospitalization. In accordance with French legislation, we were able to access data for a 10-year period between January 1, 2010, and December 31, 2019. To allow a minimum of 2 years for the detection of medical history, we excluded individuals who had a first pregnancy-associated hospitalization detected before January 1, 2012.

All patient records were screened to detect linkage anomalies between hospitalizations. Patients with unclear information regarding age, sex, or death between hospitalizations were excluded.

### Pregnancy With and Without Placental Syndromes

All female individuals aged 12 to 55 years who had their first pregnancy-associated hospitalization registered between January 1, 2012, and December 31, 2019 were included. Pregnancy-associated hospitalizations were detected through *ICD-10* codes O, Z321, Z33, Z34, Z35, Z36, Z37, and Z39. If several pregnancy-associated hospitalizations were detected, the first hospitalization was used as the reference. To account for temporality between detected hospitalization and pregnancy complications among multiple potential hospitalizations, diagnoses that occurred up to 9 months after the reference date were considered part of the same first pregnancy.

We defined the group of female patients with placental syndrome according to the diagnostic codes for eclampsia (*ICD-10* code O15) or preeclampsia (*ICD-10* codes O11 and O14) associated with the first detected pregnancy. To explore the potential associations of exposure group definitions in sensitivity analyses, we also identified diagnostic codes for abruptio placenta (*ICD-10* code O45), fetal growth restriction (*ICD-10* code O36.5), and intrauterine fetal death (*ICD-10* codes O021 and O36.4) that occurred during the first detected pregnancy.

The control group comprised female individuals who had a pregnancy-associated hospitalization without a diagnosis of preeclampsia or eclampsia during their first detected pregnancy. All individuals with a history of cancer detected before the pregnancy of interest or within 9 months after the first detected pregnancy were excluded.

### Occurrence of Cancer

New occurrences of cancer and cancer-associated blood diseases diagnosed after the first detected pregnancy were recorded. The *ICD-10* codes used for the search are listed in the eTable in the Supplement. Some disease codes, such as the types and localizations of hematologic cancer (ie, lymphomas or leukemias), were combined. In the case of multiple detected cancers, all cancers were analyzed with the time to event being the earliest detected cancer.

### Other Covariates

Comorbidities were identified before or during the first detected date of pregnancy through the primary or associated diagnosis at hospitalization using the following diagnostic codes: stroke (*ICD-10* code I6), ischemic heart disease (*ICD-10* codes I20, I21, I22, I23, I24, and I25), thromboembolic disease (*ICD-10* codes I80, I81, I82, and I74), kidney failure (*ICD-10* codes N18 and N19), obesity (*ICD-10* code E66), dyslipidemia (*ICD-10* code E78), high blood pressure (*ICD-10* codes I10, I11, I12, I13, and I15), chronic liver disease (*ICD-10* code K7), chronic obstructive pulmonary disease or asthma (*ICD-10* codes J41, J42, J43, J44, J45, and J46), and diabetes (*ICD-10* codes E10, E11, E12, E13, and E14).

In France, most health care–associated costs are paid by state insurance; however, most people purchase supplemental private insurance because some expenses are not covered. For people with low household income, state-issued supplementary insurance exists, and we used that information as a proxy for socioeconomic status.^21^

### Statistical Analysis

Baseline participant characteristics by exposure group were described. Categorical variables were calculated as frequencies and proportions, quantitative variables were reported as means and SDs, and the incidences and 95% CIs of different cancer types and myelodysplastic or myeloproliferative diseases were calculated for each exposure group.

A survival analysis using a Cox proportional hazards model was performed to compare cancer incidence between exposure groups, with time on study used as the measurement interval. The proportional hazards assumption was verified using Schoenfeld residuals. Follow-up time was calculated as the interval between the date of the first detected pregnancy and the date of the first detected cancer-associated hospitalization or December 31, 2019, whichever occurred first. To account for competitive risks, follow-up of participants who died without cancer before December 31, 2019, was censored at the date of death.^20^ Two models were tested. The first model was adjusted for age, socioeconomic status, and year of first detected pregnancy. The second model was adjusted for age, socioeconomic status, year of first detected pregnancy, and several comorbidities (obesity, high blood pressure, history of diabetes, dyslipidemia, history of kidney or liver failure, chronic obstructive pulmonary disease or asthma, and history of a cardiovascular event, including stroke, myocardial infarction, or thromboembolism). Age was fitted as a continuous variable after reviewing for linearity, and year of first detected pregnancy was fitted as a categorical variable, with each year designated as a category. Comorbidities and socioeconomic status were fitted as dichotomous variables. Hazard ratios (HRs) of the exposure group were presented with their 95% CIs.

To test possible bias and robustness of results, we performed sensitivity analyses in which the exposure group definition of placenta-mediated disease was successively extended to participants with other potential placenta-mediated complications, such as abruptio placenta (*ICD-10* code O45), intrauterine growth restriction (*ICD-10* code O36.5), and intrauterine fetal death (*ICD-10* code O021 or O36.4). Statistical analyses were performed using SAS software, version 7.1 (SAS Institute), with a 2-tailed significance threshold of α = .05.

## Results

A total of 6 343 356 female individuals with a pregnancy-associated hospitalization between January 1, 2010, and December 31, 2019, were screened (Figure). Of those, 1 989 124 individuals who had their first detected pregnancy before January 1, 2012, were excluded. Among the remaining 4 354 232 individuals, 31 262 (0.7%) were excluded because they had a cancer-associated hospitalization detected before or during the 9 months of their first detected pregnancy. After exclusions, the analysis included 4 322 970 female patients who were registered in the French hospital discharge database for a pregnancy-associated hospitalization between January 1, 2012 and December 31, 2019. The mean (SD) age at first detected pregnancy was 29.6 (6.2) years, and 176 442 participants (4.1%) had state-issued supplementary insurance. The mean (SD) follow-up period was 4.4 (2.3) years, and the maximum follow-up was 8 years; during follow-up, 29 173 individuals (0.7%) were diagnosed with cancer, and only 1639 participants (0.0379%) died.

**Figure.**
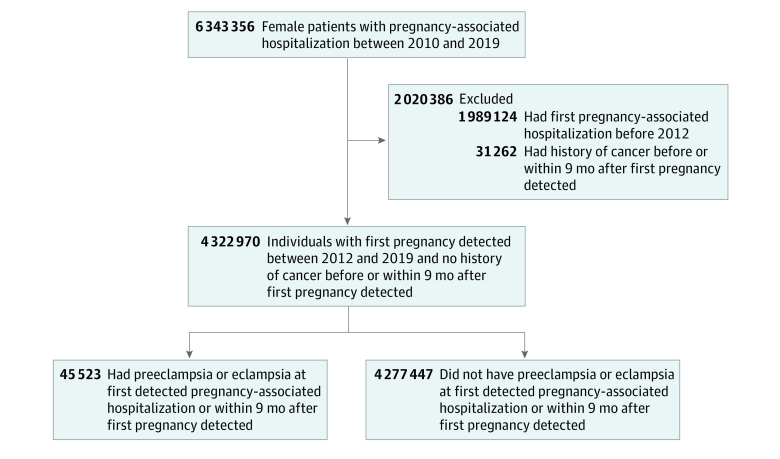
Participant Flowchart

During their first detected pregnancy, 45 523 participants (1.1%) had preeclampsia/eclampsia, representing an incidence of approximately 11 cases per 1000 pregnancies. A cumulative 196 055 person-years in the preeclampsia/eclampsia group and 19 034 861 person-years in the control group were analyzed. The age of first detected pregnancy was similar among those with and without preeclampsia/eclampsia (mean [SD], 30.0 [6.2] years and 29.0 [6.2] years, respectively) (Table 1). Compared with participants in the control group, those with preeclampsia/eclampsia had slightly higher rates of obesity (195 101 individuals [4.6%] vs 2567 individuals [5.6%]), diabetes (296 545 individuals [6.9%] vs 3618 individuals [7.9%]), high blood pressure (510 861 individuals [11.9%] vs 6112 individuals [13.4%]), dyslipidemia (152 186 individuals [3.6%] vs 1873 individuals [4.1%]), kidney failure (564 801 [13.2%] vs 6538 individuals [14.4%]), liver failure (67 548 individuals [1.6%] vs 839 individuals [1.8%]), chronic obstructive pulmonary disease or asthma (139 171 individuals [3.3%] vs 1676 individuals [3.7%]), and cardiovascular events, such as stroke, myocardial infarction, or thromboembolism (309 861 individuals [7.2%] vs 3828 individuals [8.4%]).

**Table 1.** Participant Characteristics

Characteristic	No. (%)	
	Without preeclampsia or eclampsia	With preeclampsia or eclampsia
Total participants, No.	4 277 447	45 523
Age, mean (SD), y	29.0 (6.2)	30.0 (6.2)
State-issued supplementary health insurance	174 537 (4.1)	1905 (4.2)
Health history		
Obesity	195 101 (4.6)	2567 (5.6)
Diabetes	296 545 (6.9)	3618 (7.9)
High blood pressure	510 861 (11.9)	6112 (13.4)
Dyslipidemia	152 186 (3.6)	1873 (4.1)
Cardiovascular event	309 861 (7.2)	3828 (8.4)
Kidney failure	564 801 (13.2)	6538 (14.4)
Liver failure	67 548 (1.6)	839 (1.8)
COPD or asthma	139 171 (3.3)	1676 (3.7)

Abbreviations: COPD, chronic obstructive pulmonary disease.

The overall incidence of cancer was 1.51 cases per 1000 person-years (95% CI, 1.34-1.68 cases per 1000 person-years) among participants with preeclampsia/eclampsia and 1.52 cases per 1000 person-years (95% CI, 1.50-1.54 cases per 1000 person-years) among those without preeclampsia/eclampsia (adjusted HR [AHR], 0.94; 95% CI, 0.84-1.05) (Table 2).

**Table 2.** Cancer Incidence per 1000 Person-Years and Associated Cox Proportional Hazards Ratios

Type of cancer or cancer-associated blood disease	With preeclampsia or eclampsia (n = 45 523)		Without preeclampsia or eclampsia (n = 4 277 447)		AHR (95% CI)	
	No.	Incidence (95% CI)	No.	Incidence (95% CI)	Model 1^a^	Model 2^b^
All cancers	297	1.51 (1.34-1.68)	28 876	1.52 (1.50-1.54)	0.94 (0.84-1.06)	0.94 (0.84-1.05)
Solid tumors	273	1.39 (1.22-1.56)	27 073	1.42 (1.40-1.44)	0.92 (0.82-1.04)	0.92 (0.81-1.03)
Oncohematologic disease	26	0.13 (0.08-0.18)	1977	0.10 (0.10-0.10)	1.25 (0.85-1.84)	1.25 (0.85-1.83)
Myelodysplastic or myeloproliferative syndrome	15	0.08 (0.04-0.12)	569	0.03 (0.03-0.03)	2.46 (1.47-4.11)	2.43 (1.46-4.06)
Cervical cancer	61	0.31 (0.23-0.39)	7775	0.41 (0.40-0.42)	0.75 (0.58-0.97)	0.75 (0.58-0.96)
Breast cancer	73	0.37 (0.28-0.46)	7973	0.42 (0.41-0.43)	0.79 (0.63-0.99)	0.79 (0.62-0.99)
Thyroid cancer	28	0.14 (0.09-0.19)	2478	0.13 (0.12-0.14)	1.06 (0.73-1.54)	1.06 (0.73-1.54)
Colorectal cancer	16	0.08 (0.04-0.12)	1741	0.09 (0.09-0.09)	0.81 (0.50-1.33)	0.81 (0.49-1.32)
Kidney cancer	8	0.04 (0.01-0.07)	326	0.02 (0.02-0.02)	2.21 (1.09-4.45)	2.19 (1.09-4.42)
Lung and pleural cancer	8	0.04 (0.01-0.07)	492	0.03 (0.03-0.03)	1.40 (0.70-2.82)	1.40 (0.69-2.81)
Brain and nervous system cancer	12	0.06 (0.03-0.09)	718	0.04 (0.04-0.04)	1.55 (0.88-2.71)	1.54 (0.87-2.73)
Upper aerodigestive tract cancer	6	0.03 (0.01-0.05)	399	0.02 (0.02-0.02)	1.39 (0.62-3.11)	1.38 (0.62-3.09)
Skin cancer	30	0.15 (0.10-0.20)	1985	0.10 (0.10-0.10)	1.36 (0.95-1.95)	1.36 (0.95-1.95)

Abbreviation: AHR, adjusted hazard ratio.

^a^ Model 1 was adjusted for age, insurance status, and year of first-detected pregnancy.

^b^ Model 2 was adjusted for age, insurance status, year of first-detected pregnancy, and comorbidities (obesity, high blood pressure, history of diabetes, dyslipidemia, history of kidney or liver failure, chronic obstructive pulmonary disease or asthma, and history of cardiovascular disease).

The incidence of myelodysplastic or myeloproliferative diseases was 0.08 cases per 1000 person-years (95% CI, 0.04-0.12 cases per 1000 person-years) among those with preeclampsia/eclampsia vs 0.03 cases per 1000 person-years (95% CI, 0.03-0.03 cases per 1000 person-years) among those without preeclampsia/eclampsia (AHR, 2.43; 95% CI, 1.46-4.06). Kidney cancer, although rare, was overrepresented in participants who had preeclampsia/eclampsia (AHR, 2.19; 95% CI, 1.09-4.42), and a decrease in breast cancer and cervical cancer after preeclampsia/eclampsia was observed, with AHRs of 0.79 (95% CI, 0.62-0.99) and 0.75 (95% CI, 0.58-0.96), respectively. No significant differences in the incidence of any of the other cancers examined in the study were found.

The results of the sensitivity analyses by exposure group were consistent, with some reductions in associated risk when the exposure group definitions were expanded (Table 3). Two results were noteworthy. First, the HR increased when intrauterine fetal death was included in the exposure group for myelodysplastic or myeloproliferative diseases (HR, 2.48; 95% CI, 1.75-3.53). Second, the results for breast and cervical cancer varied when the exposure group definition changed, with a decreased and nonsignificant association with breast cancer when intrauterine fetal death (HR, 0.88; 95% CI, 0.77-1.02) or abruptio placenta (HR, 0.84; 95% CI, 0.69-1.04) was added and a nonsignificant association with cervical cancer when fetal growth restriction (HR, 0.93; 95% CI, 0.78-1.10) was added.

**Table 3.** Sensitivity Analysis of Myelodysplastic or Myeloproliferative Diseases and Cancers by Exposure Group Definition^a^

Exposure group definition	Total participants, No.	Myelodysplastic or myeloproliferative disease		Breast cancer		Cervical cancer		Kidney cancer	
		No.	AHR (95% CI)	No.	AHR (95% CI)	No.	AHR (95% CI)	No.	AHR (95% CI)
Preeclampsia/ eclampsia	45 523	15	2.43 (1.46-4.06)	73	0.79 (0.62-0.99)	61	0.75 (0.58-0.96)	8	2.19 (1.09-4.42)
Preeclampsia/eclampsia and abruptio placenta	52 564	15	2.09 (1.25-3.49)	90	0.84 (0.69-1.04)	74	0.78 (0.62-0.98)	9	2.13 (1.10-4.13)
Preeclampsia/eclampsia and fetal growth restriction	79 421	24	2.32 (1.54-3.49)	115	0.76 (0.63-0.92)	129	0.93 (0.78-1.10)	12	1.99 (1.12-3.55)
Preeclampsia/eclampsia and intrauterine fetal death	93 321	33	2.48 (1.75-3.53)	192	0.88 (0.77-1.02)	140	0.79 (0.67-0.94)	14	1.70 (0.99-2.90)
Preeclampsia/eclampsia, abruptio placenta, fetal growth restriction, and intrauterine fetal death	134 260	42	2.29 (1.67-3.14)	251	0.87 (0.77-0.99)	221	0.89 (0.78-1.02)	19	1.71 (1.07-2.71)

Abbreviation: AHR, adjusted hazard ratio.

^a^ Results from Cox proportional hazards model adjusted for age, insurance status, year of first detected pregnancy, and comorbidities (obesity, high blood pressure, history of diabetes, dyslipidemia, history of kidney or liver failure, chronic obstructive pulmonary disease or asthma, and history of cardiovascular disease).

## Discussion

### Main Findings

This population-wide cohort study did not find an overall increase in the rate of cancer diagnosis after a first pregnancy complicated by preeclampsia/eclampsia. However, an analysis by type of cancer indicated a significant excess in the incidence of chronic myeloproliferative and myelodysplastic syndromes and kidney cancer among participants with preeclampsia/eclampsia during their first pregnancy. These associations remained in the sensitivity analyses. For kidney cancer, the HR decreased as expected when the exposure group definition was expanded, suggesting that the observed increased incidence was associated with a preeclampsia/eclampsia mechanism. For myelodysplastic and myeloproliferative diseases, the HR increased when intrauterine fetal death was included in the exposure group. This finding suggests a possible common factor between preeclampsia/eclampsia and some of the intrauterine fetal death mechanisms, which may have produced an increased incidence of myelodysplastic or myeloproliferative diseases.

For breast cancer, a small protective benefit was found among those with preeclampsia/eclampsia; however, this benefit disappeared when the exposure group definition was expanded to include either abruptio placenta or intrauterine fetal death but remained when fetal growth restriction was included. For cervical cancer, the protective benefit disappeared when the exposure group definition was expanded to fetal growth restriction.

The incidence of preeclampsia/eclampsia in this data set was consistent with reference data for the French population, with estimates ranging from 1% to 3% in nulliparous individuals and 0.5% to 1.5% in multiparous individuals.^22^ A meta-analysis performed in 2007^8^ found no association between preeclampsia and overall cancer risk after an average weighted follow-up of 13.9 years. The data from the present study, which had a shorter follow-up period, were consistent with these findings. A 2018 meta-analysis of cohort studies^18^ did not find an association between preeclampsia and a maternal risk of breast cancer, even if an increased risk existed among specific populations, as reported in the Jerusalem Perinatal Study.^23^ However, preeclampsia was associated with a significantly lower maternal incidence of breast cancer among those who delivered male offspring.^18^ The present study could not examine the association of offspring sex.

The current study’s findings are also consistent with those of the Generations Study,^11^ a large prospective study in the United Kingdom that found a decreased breast cancer risk among premenopausal participants who had experienced preeclampsia, as well as the results of a large population-based case-control study of female patients with breast cancer in Scandinavia.^24^ To our knowledge, the present study is the first to explore the association of preeclampsia/eclampsia with chronic myeloproliferative or myelodysplastic diseases and kidney cancer.

### Pathophysiological Hypotheses

Placental diseases can produce acute kidney injury, and this injury can lead to chronic arterial hypertension, which is an independent risk factor for kidney cell carcinoma.^25^ Although hypertensive disorders of pregnancy, including preeclampsia/eclampsia, are known to be associated with increases in the risk of stroke, coronary artery disease, cardiac arrhythmias, chronic kidney disease, and multimorbidity,^26^ creating an opportunity for cardiovascular risk management,^27^ the data from the present study could reflect the consequences of female hypertension, which may have produced an increased risk of kidney cell carcinoma.

The biological mechanisms underlying the associations with placental diseases and chronic myeloproliferative and myelodysplastic syndromes remain unclear. Although sporadic myelodysplasia is primarily diagnosed in older adults, myelodysplasia in young and middle-aged adults is frequently associated with genetic syndromes,^28^ none of which have been described as being associated with exposure to placental diseases during pregnancy. Data regarding the association between Philadelphia-negative chronic myeloproliferative neoplasms and pregnancy mainly pertain to essential thrombocythemia and suggest substantial maternal morbidity, including preeclampsia/eclampsia, intrauterine growth restriction, stillbirth, and premature delivery.^29^ These disorders are associated with acquired variations in the Janus kinase 2 (*JAK2*), calcitonin (*CALC*), or MPL protooncogene, thrombopoietin receptor (*MPL*) genes, which have not been described in those with preeclampsia/eclampsia. A latent underlying and undiagnosed form of these disorders in some placental disease cases cannot be excluded.

Another possibility is that the substantial biological stress associated with the onset and progression of placental diseases may, in rare cases, induce acquired variations in the somatic DNA (eg, genes that define clonal hematopoiesis of indeterminate potential, which alone precipitates subsequent myelodysplastic syndrome).^30^ Of note, clear associations have been described between clonal hematopoiesis of indeterminate potential, atherothrombosis, and cancer^31,32^; a previous study reported an excess of *JAK2* V617F variations among individuals experiencing unexplained fetal loss during their first pregnancy.^33^ Among biological stresses, inflammasome activation is a signature of preeclampsia/eclampsia,^34,35^ and in hematopoietic stem or progenitor cells, inflammasome activation has been implicated as an important convergence signal in myelodysplastic syndromes with consequent clonal expansion.^36^ Inflammatory cytokines and mediators are also suspected to have an important role in the initiation, propagation, and progression of myelodysplastic syndromes.^37^

Endothelial progenitor cell dysfunction is a component of the defective vascular niche associated with myelodysplasia^38^ and may be the common factor between preeclampsia/eclampsia and increased risk of cardiovascular and myelodysplastic diseases. With regard to breast cancer, it has been hypothesized that an individual’s hormonal profile^24,39^ and the persistence of an antiangiogenic profile^24,40^ could explain the lower incidence observed among those with a history of preeclampsia/eclampsia. Given that a normal pregnancy induces immunological tolerance, it has been suggested that preeclampsia could be associated with an abnormal immune response to the fetus.^24,41^ This possibility may therefore suggest a more aggressive immune response to a developing tumor. Although speculative, these hypotheses could also explain the lower incidence of cervical cancer observed.

### Strengths and Limitations

This study has strengths. It used a register of pregnancies and cancers that was almost exhaustive. Large numbers of cancer diagnoses were collected, which provided sufficient statistical power for analyses of cancer types, including rare cancers. In France, most women deliver in a maternity hospital (in 2016, only 0.6% of all deliveries occurred outside a maternity unit or hospital).^20^ Placental diseases are defined using objective criteria with clear definitions that are collegially applied by obstetricians; the coding of placental diseases is therefore assumed to be correct. The first national validation study of many perinatal algorithms also suggested that the French national hospital database is an appropriate data source for epidemiological studies.^42^ In addition, because most cancers require hospitalization, we can also be confident in the completeness of the outcome data collected. Although the study population was young, the study accounted for potential competitive risk among participants without cancer who died while hospitalized by censoring at the date of death (only 0.0379% of participants in the cohort died during follow-up, and approximately two-thirds of deaths in France occur in a medical facility^43^). Therefore, factors that may be associated with the risk of death were unlikely to have had substantial consequences for our results.^44^

This study also has limitations. The database used in the study limited the assessment of some important information in the field of cancer risk, such as family history, lifestyle habits, hormone-based treatments, some pregnancy-associated information, and exposure to environmental carcinogens, which were not available and may be confounding factors. Bias in the collection of comorbidities also remains possible because we could only access information registered during the hospital stay, and some comorbidities do not require hospitalization, which could have limited the quality of our adjustments. It is also possible that the first pregnancy detected was not actually the first pregnancy for some participants, which would result in a limited number of multiparous participants in the analysis. This limitation may have had a small impact with regard to some cancer incidence estimates. Although the incidence of some cancers may be associated with the severity of placental disease,^16^ these data, as well as information on placental disease subtypes, were not available. In addition, some differences may appear over time, and we cannot comment on cancer risks in the long-term.

## Conclusions

Among pregnant individuals in the French hospital discharge database from 2012 to 2019, an increased incidence of myelodysplastic or myeloproliferative neoplasms and kidney cancers and a slightly decreased incidence of breast and cervical cancers were observed among those with preeclampsia/eclampsia during their first pregnancy. These results persisted when the spectrum of placental diseases was analyzed and suggest the presence of shared pathophysiological factors to be explored in the future.

## Notes

Supplement. **eTable.** *ICD-10* Codes Used for Cancer Detection

## References

[1] Abalos E, Cuesta C, Grosso AL, Chou D, Say L. Global and regional estimates of preeclampsia and eclampsia: a systematic review. Eur J Obstet Gynecol Reprod Biol. 2013;170(1):1-7. doi:10.1016/j.ejogrb.2013.05.005 23746796

[2] Burton GJ, Redman CW, Roberts JM, Moffett A. Pre-eclampsia: pathophysiology and clinical implications. *BMJ*. Published online July 15, 2019. doi:10.1136/bmj.l2381 31307997

[3] Rana S, Lemoine E, Granger JP, Karumanchi SA. Preeclampsia: pathophysiology, challenges, and perspectives. Circ Res. 2019;124(7):1094-1112. doi:10.1161/CIRCRESAHA.118.313276 30920918

[4] Staff AC. The two-stage placental model of preeclampsia: an update. J Reprod Immunol. 2019;134-135:1-10. doi:10.1016/j.jri.2019.07.004 31301487

[5] Brosens I, Pijnenborg R, Vercruysse L, Romero R. The “great obstetrical syndromes” are associated with disorders of deep placentation. Am J Obstet Gynecol. 2011;204(3):193-201. doi:10.1016/j.ajog.2010.08.009 21094932 PMC3369813

[6] Williams D. Pregnancy: a stress test for life. Curr Opin Obstet Gynecol. 2003;15(6):465-471. doi:10.1097/00001703-200312000-00002 14624211

[7] Jonsdottir LS, Arngrimsson R, Geirsson RT, Sigvaldason H, Sigfusson N. Death rates from ischemic heart disease in women with a history of hypertension in pregnancy. Acta Obstet Gynecol Scand. 1995;74(10):772-776. doi:10.3109/00016349509021195 8533558

[8] Bellamy L, Casas JP, Hingorani AD, Williams DJ. Pre-eclampsia and risk of cardiovascular disease and cancer in later life: systematic review and meta-analysis. BMJ. 2007;335(7627):974. doi:10.1136/bmj.39335.385301.BE 17975258 PMC2072042

[9] Nechuta S, Paneth N, Velie EM. Pregnancy characteristics and maternal breast cancer risk: a review of the epidemiologic literature. Cancer Causes Control. 2010;21(7):967-989. doi:10.1007/s10552-010-9524-7 20224871 PMC3863387

[10] Kim KJ, Cho CS, Kim WU. Role of placenta growth factor in cancer and inflammation. Exp Mol Med. 2012;44(1):10-19. doi:10.3858/emm.2012.44.1.023 22217448 PMC3277893

[11] Wright LB, Schoemaker MJ, Jones ME, Ashworth A, Swerdlow AJ. Breast cancer risk in relation to history of preeclampsia and hyperemesis gravidarum: prospective analysis in the Generations Study. Int J Cancer. 2018;143(4):782-792. doi:10.1002/ijc.31364 29516507 PMC6055869

[12] Pacheco NLP, Andersen AMN, Kamper-Jorgensen M. Preeclampsia and breast cancer: the influence of birth characteristics. Breast. 2015;24(5):613-617. doi:10.1016/j.breast.2015.06.006 26144638

[13] Yang H, He W, Eriksson M, . Inherited factors contribute to an inverse association between preeclampsia and breast cancer. Breast Cancer Res. 2018;20(1):6. doi:10.1186/s13058-017-0930-6 29361985 PMC5782395

[14] Trabert B, Troisi R, Grotmol T, . Associations of pregnancy-related factors and birth characteristics with risk of endometrial cancer: a Nordic population-based case-control study. Int J Cancer. 2020;146(6):1523-1531. doi:10.1002/ijc.32494 31173648 PMC6898733

[15] Cornish R, Staff AC, Boyd A, . Maternal reproductive hormones and angiogenic factors in pregnancy and subsequent breast cancer risk. Cancer Causes Control. 2019;30(1):63-74. doi:10.1007/s10552-018-1100-6 30506491 PMC6438198

[16] Hallum S, Pinborg A, Kamper-Jorgensen M. Long-term impact of preeclampsia on maternal endometrial cancer risk. Br J Cancer. 2016;114(7):809-812. doi:10.1038/bjc.2016.55 26964032 PMC4984869

[17] Behrens I, Basit S, Jensen A, . Hypertensive disorders of pregnancy and subsequent risk of solid cancer—a nationwide cohort study. Int J Cancer. 2016;139(1):58-64. doi:10.1002/ijc.30065 26919086

[18] Sun M, Fan Y, Hou Y, Fan Y. Preeclampsia and maternal risk of breast cancer: a meta-analysis of cohort studies. J Matern Fetal Neonatal Med. 2018;31(18):2484-2491. doi:10.1080/14767058.2017.1342806 28715959

[19] Boudemaghe T, Belhadj I. Data resource profile: the French National Uniform Hospital Discharge Data Set Database (PMSI). Int J Epidemiol. 2017;46(2):392-392d. doi:10.1093/ije/dyw359 28168290

[20] Bellamy V. The 784 000 births of 2016 took place in 2800 villages. National Institute of Statistics and Economic Studies. August 30, 2017. Accessed August 31, 2020. https://insee.fr/fr/statistiques/3047024.

[21] Republique Francaise. Supplemental solidarity-based health (ex-CMU-C). Service-Public.fr website. Updated April 1, 2021. Accessed August 31, 2020. https://www.service-public.fr/particuliers/vosdroits/F10027.

[22] Goffinet F. Epidémiologie. Ann Fr Anesth Reanim. 2010;29(3):e7-e12. doi:10.1016/j.annfar.2010.02.010 20338718

[23] Calderon-Margalit R, Friedlander Y, Yanetz R, . Preeclampsia and subsequent risk of cancer: update from the Jerusalem Perinatal Study. Am J Obstet Gynecol. 2009;200(1):63.e1-63.e5. doi:10.1016/j.ajog.2008.06.057 18822400 PMC2660849

[24] Troisi R, Gulbech Ording A, Grotmol T, . Pregnancy complications and subsequent breast cancer risk in the mother: a Nordic population-based case-control study. Int J Cancer. 2018;143(8):1904-1913. doi:10.1002/ijc.31600 29752724 PMC6128759

[25] Capitanio U, Bensalah K, Bex A, . Epidemiology of renal cell carcinoma. Eur Urol. 2019;75(1):74-84. doi:10.1016/j.eururo.2018.08.036 30243799 PMC8397918

[26] Garovic VD, White WM, Vaughan L, . Incidence and long-term outcomes of hypertensive disorders of pregnancy. J Am Coll Cardiol. 2020;75(18):2323-2334. doi:10.1016/j.jacc.2020.03.028 32381164 PMC7213062

[27] Smith GN, Louis JM, Saade GR. Pregnancy and the postpartum period as an opportunity for cardiovascular risk identification and management. Obstet Gynecol. 2019;134(4):851-862. doi:10.1097/AOG.0000000000003363 31503139

[28] Babushok DV, Bessler M. Genetic predisposition syndromes: when should they be considered in the work-up of MDS? Best Pract Res Clin Haematol. 2015;28(1):55-68. doi:10.1016/j.beha.2014.11.004 25659730 PMC4323616

[29] Robinson SE, Harrison CN. How we manage Philadelphia-negative myeloproliferative neoplasms in pregnancy. Br J Haematol. 2020;189(4):625-634. doi:10.1111/bjh.16453 32150650

[30] Sperling AS, Gibson CJ, Ebert BL. The genetics of myelodysplastic syndrome: from clonal haematopoiesis to secondary leukaemia. Nat Rev Cancer. 2017;17(1):5-19. doi:10.1038/nrc.2016.112 27834397 PMC5470392

[31] Jaiswal S, Fontanillas P, Flannick J, . Age-related clonal hematopoiesis associated with adverse outcomes. N Engl J Med. 2014;371(26):2488-2498. doi:10.1056/NEJMoa1408617 25426837 PMC4306669

[32] Jaiswal S, Natarajan P, Silver AJ, . Clonal hematopoiesis and risk of atherosclerotic cardiovascular disease. N Engl J Med. 2017;377(2):111-121. doi:10.1056/NEJMoa1701719 28636844 PMC6717509

[33] Mercier E, Lissalde-Lavigne G, Gris JC. JAK2 V617F mutation in unexplained loss of first pregnancy. N Engl J Med. 2007;357(19):1984-1985. doi:10.1056/NEJMc071528 17989398

[34] Kohli S, Ranjan S, Hoffmann J, . Maternal extracellular vesicles and platelets promote preeclampsia via inflammasome activation in trophoblasts. Blood. 2016;128(17):2153-2164. doi:10.1182/blood-2016-03-705434 27589872

[35] Romao-Veiga M, Matias ML, Ribeiro VR, . Induction of systemic inflammation by hyaluronan and hsp70 in women with pre-eclampsia. Cytokine. 2018;105:23-31. doi:10.1016/j.cyto.2018.02.007 29438905

[36] Sallman DA, Cluzeau T, Basiorka AA, List A. Unraveling the pathogenesis of MDS: the NLRP3 inflammasome and pyroptosis drive the MDS phenotype. Front Oncol. 2016;6:151. doi:10.3389/fonc.2016.00151 27379212 PMC4909736

[37] Banerjee T, Calvi LM, Becker MW, Liesveld JL. Flaming and fanning: the spectrum of inflammatory influences in myelodysplastic syndromes. Blood Rev. 2019;36:57-69. doi:10.1016/j.blre.2019.04.004 31036385 PMC6711159

[38] Teofili L, Martini M, Nuzzolo ER, . Endothelial progenitor cell dysfunction in myelodysplastic syndromes: possible contribution of a defective vascular niche to myelodysplasia. Neoplasia. 2015;17(5):401-409. doi:10.1016/j.neo.2015.04.001 26025663 PMC4468365

[39] Cnattingius S, Torrang A, Ekbom A, Granath F, Petersson G, Lambe M. Pregnancy characteristics and maternal risk of breast cancer. JAMA. 2005;294(19):2474-2480. doi:10.1001/jama.294.19.2474 16287958

[40] Baumwell S, Karumanchi SA. Pre-eclampsia: clinical manifestations and molecular mechanisms. Nephron Clin Pract. 2007;106(2):c72-c81. doi:10.1159/000101801 17570933

[41] Polednak AP. Pre-eclampsia, autoimmune diseases and breast cancer etiology. Med Hypotheses. 1995;44(5):414-418. doi:10.1016/0306-9877(95)90271-6 8583975

[42] Goueslard K, Cottenet J, Benzenine E, Tubert-Bitter P, Quantin C. Validation study: evaluation of the metrological quality of French hospital data for perinatal algorithms. BMJ Open. 2020;10(5):e035218. doi:10.1136/bmjopen-2019-035218 32404391 PMC7228531

[43] Number of daily deaths: France, regions and departments. National Institute of Statistics and Economic Studies. May 7, 2021. Accessed August 31, 2020. https://www.insee.fr/fr/statistiques/4500439?sommaire=4487854.

[44] Austin PC, Lee DS, Fine JP. Introduction to the analysis of survival data in the presence of competing risks. Circulation. 2016;133(6):601-609. doi:10.1161/CIRCULATIONAHA.115.017719 26858290 PMC4741409

