# Supplementary material for: Assessment of All-Cause Cancer Incidence Among Individuals With Preeclampsia or Eclampsia During First Pregnancy
Source: JAMA Netw Open. 2021 Jun 23;4(6):e2114486. doi: 10.1001/jamanetworkopen.2021.14486 (PMC8223101; doi:10.1001/jamanetworkopen.2021.14486)
Supplement: Supplement. — eTable. ICD-10 Codes Used for Cancer Detection [file jamanetwopen-e2114486-s001.pdf]

Supplementary Online Content

Serrand C, Mura T, Fabbro-Peray P, et al. Assessment of all-cause cancer incidence among individuals with preclampsia or eclampsia during first pregnancy. *JAMA Netw Open*. 2021;4(6):e2114486. doi:10.1001/jamanetworkopen.2021.14486

eTable. *ICD-10* Codes Used for Cancer Detection

This supplementary material has been provided by the authors to give readers additional information about their work.

© 2021 Serrand C et al. *JAMA Network Open*.

eTable. *ICD-10* Codes Used for Cancer Detection

Cancer grouping	ICD-10 Codes
**Overall**	C39 C38 C388 C39 C390 C398 C399 C4672 D02<br>D023 D024 D38 D385 D386 C75 C750 C751 C752<br>C753 C754 C755 C758 C759 D093 D44 D442 D443<br>D444 D4440 D4448 D445 D446 D447 D448 D449 C94<br>C940 C941 C942 C943 C944 C945 C947 C95 C950<br>C951 C952 C957 C959 C21 C210 C211 C212 C218<br>C26 C260 C261 C268 C269 C4671 D01 D013 D017<br>D019 D37 D377 D379 C51 C510 C511 C512 C518<br>C519 C52 C57 C578 C579 D071 D072 D073 D39<br>D397 D399 C96 C960 C961 C962 C963 C964 C965<br>C966 C967 C968 C969 C70 C700 C701 C709 C71<br>C710 C711 C712 C713 C714 C715 C716 C717 C718<br>C719 D42 D420 D421 D429 D430 D431 D432 C53<br>C530 C531 C538 C539 D06 D060 D061 D067 D069<br>C54 C540 C541 C542 C543 C548 C549 C55 D070<br>C18 C180 C181 C182 C183 C184 C185 C186 C187<br>C188 C189 C189+0 C189+8 C19 C20 D010 D011 D012<br>D373 D374 D375 C16 C160 C161 C162 C163 C164<br>C165 C166 C168 C169 C169+0 C169+8 C17 C170 C171<br>C172 C173 C178 C179 D00 D002 D014 D371 D372<br>C22 C220 C221 C222 C223 C224 C227 C229 C23<br>C24 C240 C241 C248 C249 D015 D376 C30 C300<br>C301 C31 C310 C311 C312 C313 C318 C319 C32<br>C320 C321 C322 C323 C328 C329 D020 D380 C91<br>C910 C911 C912 C913 C914 C915 C916 C917 C918<br>C919 C93 C930 C931 C932 C933 C937 C939 C92<br>C920 C921 C922 C923 C924 C925 C926 C927 C928<br>C929 C81 C810 C811 C812 C813 C814 C817 C819<br>C82 C820 C821 C822 C823 C824 C825 C826 C827<br>C829 C83 C830 C831 C832 C833 C834 C835 C836<br>C837 C838 C839 C84 C840 C841 C842 C843 C844<br>C845 C846 C847 C848 C849 C85 C850 C851 C852<br>C857 C859 C86 C860 C861 C862 C863 C864 C865<br>C866 C00 C000 C001 C002 C003 C004 C005 C006<br>C008 C009 C01 C02 C020 C021 C022 C023 C024<br>C028 C029 C03 C030 C031 C039 C04 C040 C041<br>C048 C049 C05 C050 C051 C052 C058 C059 C06<br>C060 C061 C062 C068 C069 C07 C08 C080 C081<br>C088 C089 C09 C090 C091 C098 C099 C10 C100<br>C101 C102 C103 C104 C108 C109 C11 C110 C111<br>C112 C113 C118 C119 C12 C13 C130 C131 C132<br>C138 C139 C14 C140 C141 C142 C148 C462 D000<br>D370 C88 C880 C881 C882 C883 C884 C887 C889<br>C946 D45 D46 D460 D461 D462 D463 D464 D465<br>D466 D467 D469 D47 D470 D471 D472 D473 D474<br>D475 D477 D479 C720 C721 C722 C723 C724 C725<br>D433 D434 C90 C900 C901 C902 C903 C43 C430<br>C431 C432 C433 C434 C435 C436 C437 C438 C439<br>D03 D030 D031 D032 D033 D034 D035 D036 D037<br>D038 D039 C47 C470 C471 C472 C473 C474 C475<br>C476 C478 C479 D482 C15 C150 C151 C152 C153<br>C154 C155 C158 C159 D001 C69 C690 C691 C692<br>C693 C694 C695 C696 C698 C699 D092 C40 C400<br>C401 C402 C403 C408 C409 C41 C410 C411 C412<br>C413 C414 C418 C419 D480 C56 C570 C571 C572<br>C573 C574 C577 D391 C25 C250 C251 C252 C253

© 2021 Serrand C et al. *JAMA Network Open*.

		C254 C254+0 C254+8 C257 C258 C259 C259+0 C259+8 C44<br>C440 C441 C442 C443 C444 C445 C446 C447 C448<br>C449 C460 D04 D040 D041 D042 D043 D044 D045<br>D046 D047 D048 D049 D485 C58 D392 C384 C450<br>D382 C64 C65 D410 D411 C451 C48 C480 C481<br>C482 C488 D483 D4830 D4838 D484 C463 C50 C500<br>C501 C502 C503 C504 C505 C506 C508 C509 D05<br>D050 D051 D057 D059 D486 C762 C760 C764 C765<br>C76 C767 C768 C761 C80 C800 C809 D09 D097<br>D099 D48 D487 D489 C74 C740 C741 C749 D441<br>C72 C728 C729 D43 D437 D439 C37 C380 C381<br>C382 C383 C452 D383 D384 C73 D440 C45 C457<br>C459 C46 C461 C467 C4678 C468 C469 C49 C490<br>C491 C492 C493 C4930 C4938 C494 C4940 C4948 C495<br>C4950 C4958 C496 C498 C499 D481 C33 C34 C340<br>C341 C342 C343 C348 C349 D021 D022 D381<br>C80+0 C97 D390 C4670 C66 C67 C670 C671 C672<br>C673 C674 C675 C676 C677 C678 C679 C68 C680<br>C681 C688 C689 D090 D091 D41 D412 D413 D414<br>D417 D419
**Solid tumors**		C38 C388 C39 C390 C398 C399 C4672 D02 D023<br>D024 D38 D385 D386 C75 C750 C751 C752 C753<br>C754 C755 C758 C759 D093 D44 D442 D443 D444<br>D4440 D4448 D445 D446 D447 D448 D449 C21 C210<br>C211 C212 C218 C26 C260 C261 C268 C269<br>C4671 D01 D013 D017 D019 D37 D377 D379 C51<br>C510 C511 C512 C518 C519 C52 C57 C578 C579<br>D071 D072 D073 D39 D397 D399 C70 C700 C701<br>C709 C71 C710 C711 C712 C713 C714 C715 C716<br>C717 C718 C719 D42 D420 D421 D429 D430 D431<br>D432 C53 C530 C531 C538 C539 D06 D060 D061<br>D067 D069 C54 C540 C541 C542 C543 C548 C549<br>C55 D070 C18 C180 C181 C182 C183 C184 C185<br>C186 C187 C188 C189 C189+0 C189+8 C19 C20 D010<br>D011 D012 D373 D374 D375 C16 C160 C161 C162<br>C163 C164 C165 C166 C168 C169 C169+0 C169+8 C17<br>C170 C171 C172 C173 C178 C179 D00 D002 D014<br>D371 D372 C22 C220 C221 C222 C223 C224 C227<br>C229 C23 C24 C240 C241 C248 C249 D015 D376<br>C30 C300 C301 C31 C310 C311 C312 C313 C318<br>C319 C32 C320 C321 C322 C323 C328 C329 D020<br>D380 C00 C000 C001 C002 C003 C004 C005 C006<br>C008 C009 C01 C02 C020 C021 C022 C023 C024<br>C028 C029 C03 C030 C031 C039 C04 C040 C041<br>C048 C049 C05 C050 C051 C052 C058 C059 C06<br>C060 C061 C062 C068 C069 C07 C08 C080 C081<br>C088 C089 C09 C090 C091 C098 C099 C10 C100<br>C101 C102 C103 C104 C108 C109 C11 C110 C111<br>C112 C113 C118 C119 C12 C13 C130 C131 C132<br>C138 C139 C14 C140 C141 C142 C148 C462 D000<br>D370 C720 C721 C722 C723 C724 C725 D433 D434<br>C43 C430 C431 C432 C433 C434 C435 C436 C437<br>C438 C439 D03 D030 D031 D032 D033 D034 D035<br>D036 D037 D038 D039 47 C470 C471 C472 C473<br>C474 C475 C476 C478 C479 D482 C15 C150 C151<br>C152 C153 C154 C155 C158 C159 D001 C69 C690<br>C691 C692 C693 C694 C695 C696 C698 C699 D092<br>C40 C400 C401 C402 C403 C408 C409 C41 C410<br>C411 C412 C413 C414 C418 C419 D480 C56 C570<br>C571 C572 C573 C574 C577 D391 C25 C250 C251

© 2021 Serrand C et al. *JAMA Network Open*.

		C252 C253 C254 C254+0 C254+8 C257 C258 C259 C259+0 C259+8 C44 C440 C441 C442 C443 C444 C445 C446 C447 C448 C449 C460 D04 D040 D041 D042 D043 D044 D045 D046 D047 D048 D049 D485 C58 D392 C384 C450 D382 C64 C65 D410 D411 C451 C48 C480 C481 C482 C488 D483 D4830 D4838 D484 C463 C50 C500 C501 C502 C503 C504 C505 C506 C508 C509 D05 D050 D051 D057 D059 D486 C762 C760 C764 C765 C76 C767 C768 C761 C80 C800 C809 D09 D097 D099 D48 D487 D489 C74 C740 C741 C749 D441 C72 C728 C729 D43 D437 D439 C37 C380 C381 C382 C383 C452 D383 D384 C73 D440 C45 C457 C459 C46 C461 C467 C4678 C468 C469 C49 C490 C491 C492 C493 C4930 C4938 C494 C4940 C4948 C495 C4950 C4958 C496 C498 C499 D481 C33 C34 C340 C341 C342 C343 C348 C349 D021 D022 D381 C80+0 C97 D390 C4670 C66 C67 C670 C671 C672 C673 C674 C675 C676 C677 C678 C679 C68 C680 C681 C688 C689 D090 D091 D41 D412 D413 D414 D417 D419
**Hematological**		C94 C940 C941 C942 C943 C944 C945 C947 C95 C950 C951 C952 C957 C959 C96 C960 C961 C962 C963 C964 C965 C966 C967 C968 C969 C91 C910 C911 C912 C913 C914 C915 C916 C917 C918 C919 C93 C930 C931 C932 C933 C937 C939 C92 C920 C921 C922 C923 C924 C925 C926 C927 C928 C929 C81 C810 C811 C812 C813 C814 C817 C819 C82 C820 C821 C822 C823 C824 C825 C826 C827 C829 C88 C880 C881 C882 C883 C884 C887 C889 C946 D45 D46 D460 D461 D462 D463 D464 D465 D466 D467 D469 D47 D470 D471 D472 D473 D474 D475 D477 D479 C90 C900 C901 C902 C903
**Myelodysplastic or myeloproliferative**		C946 D45 D46 D460 D461 D462 D463 D464 D465 D466 D467 D469 D47 D470 D471 D472 D473 D474 D475 D477 D479
**Cervical cancers**		C53 C530 C531 C538 C539 D06 D060 D061 D067 D069
**Breast cancers**		C50 C500 C501 C502 C503 C504 C505 C506 C508 C509 D05 D050 D051 D057 D059 D486
**Thyroid cancer**		C73 D440
**Colorectal cancers**		C18 C180 C181 C182 C183 C184 C185 C186 C187 C188 C189 C189+0 C189+8 C19 C20 D010 D011 D012 D373 D374 D375
**Kidney cancers**		C64 C65 D410 D411
**Lung and pleural cancers**		C384 C450 D382 C33 C34 C340 C341 C342 C343 C348 C349 D021 D022 D381 C38 C388 C39 C390 C398 C399 C4672 D02 D023 D024 D38 D385 D386
**Brain and nervous system cancers**		C70 C700 C701 C709 C71 C710 C711 C712 C713 C714 C715 C716 C717 C718 C719 D42 D420 D421 D429 D430 D431 D432 C720 C721 C722 C723 C724 C725 D433 D434 C47 C470 C471 C472 C473 C474 C475 C476 C478 C479 D482 C69 C690 C691 C692 C693 C694 C695 C696 C698 C699 D092
**Otolaryngeal and upper aero-digestive tract cancers**		C30 C300 C301 C31 C310 C311 C312 C313 C318 C319 C32 C320 C321 C322 C323 C328 C329 D020 D380 C00 C000 C001 C002 C003 C004 C005 C006 C008 C009 C01 C02 C020 C021 C022 C023 C024 C028 C029 C03 C030 C031 C039 C04 C040 C041 C048 C049 C05 C050 C051 C052 C058 C059 C06 C060 C061 C062 C068 C069 C07 C08 C080 C081

© 2021 Serrand C et al. *JAMA Network Open*.

		C088 C089 C09 C090 C091 C098 C099 C10 C100 C101 C102 C103 C104 C108 C109 C11 C110 C111 C112 C113 C118 C119 C12 C13 C130 C131 C132 C138 C139 C14 C140 C141 C142 C148 C462 D000 D370 C4670
**Skin cancers**		C44 C440 C441 C442 C443 C444 C445 C446 C447 C448 C449 C460 D04 D040 D041 D042 D043 D044 D045 D046 D047 D048 D049 D485 C43 C430 C431 C432 C433 C434 C435 C436 C437 C438 C439 D03 D030 D031 D032 D033 D034 D035 D036 D037 D038 D039

© 2021 Serrand C et al. *JAMA Network Open*.
